# The Prebiotic Effect of Kaempferol in Regulating Bile Acid Metabolism

**DOI:** 10.1002/fsn3.70023

**Published:** 2025-02-24

**Authors:** Xiaoyan Li, Guoxin Huang, Imran Khan, Zhishan Ding, Wen Luan Wendy Hsiao, Zhongqiu Liu

**Affiliations:** ^1^ School of Medical Technology and Information Engineering Zhejiang Chinese Medical University Hangzhou Zhejiang China; ^2^ International Institute for Translational Chinese Medicine Guangzhou University of Chinese Medicine Guangzhou Guangdong China; ^3^ Clinical Research Center Shantou Central Hospital Shantou China; ^4^ Department of Biotechnology, Faculty of Chemical and Life Sciences Abdul Wali Khan University Mardan Mardan Khyber Pakhtunkhwa Pakistan; ^5^ State Key Laboratory of Quality Research in Chinese Medicine Macau University of Science and Technology Macau China

**Keywords:** bile acid, farnesoid X receptor, gut microbiota, kaempferol, prebiotic

## Abstract

Kaempferol (Kae), as a homologous flavonoid, plays a pivotal role in human nutrition and disease treatment. This study endeavors to elucidate the in vivo metabolism of Kae and its potential to modulate the interplay between bile acids (BAs) and gut microbiota (GM). After Kae administration, we analyzed pharmacokinetics, BA levels, and drug metabolic enzymes (DMEs) amount using LC–MS/MS. Subsequently, we checked the gene and protein expression with qRT‐PCR and western blot and studied the changes in GM using 16S rRNA sequencing, accompanying in‐depth data analysis. Finally, molecular docking was employed to explore Kae's interaction with the Farnesoid X receptor (FXR). Kae enhances its own absorption and metabolic circulation in vivo by upregulating the UDP‐Glucuronosyltransferases (UGTs) expression. Furthermore, Kae significantly suppressed the expression of cholesterol 7α‐hydroxylase (CYP7A1) while concurrently elevating the sterol 27‐hydroxylase (CYP27A1) expression, by activating the liver FXR, a nuclear transcription factor involved in the regulation of CYPs and UGTs enzymes. For BA analysis, Kae induced the upregulation of tauro‐BAs by attenuating the activity of bile salt hydrolases (BSH), which correlated with shifts in the GM composition. Specifically, Kae increased the abundance of beneficial bacteria such as *Bacteroides acidifaciens* and *Bifidobacterium choerinum* , while reduced populations of species associated with BSH deconjugation. The study indicates that Kae may serve as a prebiotic, modulating the BA‐GM interaction to confer nutritional and therapeutic advantages.

## Graphical abstract

Kaempferol (Kae) can suppress the expression of CYP7A1, while increasing the CYP27A1 and UGTs expression by activating the liver FXR. Kae modulates the BA profile by decreasing the BSH activities. Kae markedly increases the abundance of beneficial bacteria and inhibits the growth of pathogenic bacteria.

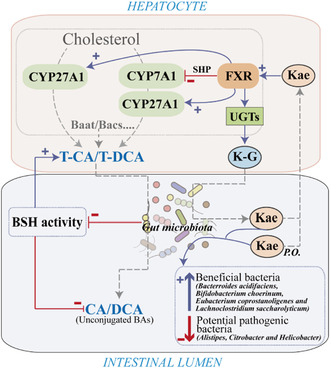

**Abbreviations**
BA: Bile acid
BAAT: Bile acid‐CoA:Amino acid N‐acyltransferase
BACS: Bile acid‐CoA ligase
BSH: Bile salt hydrolases
CA: Cholic acid
CDCA: Chenodeoxycholic acid
CDO1: Cysteine dioxygenase type 1
CSAD: Cysteine sulfinic acid decarboxylase
CYP27A1: Sterol 27‐hydroxylase
CYP7A1: Cholesterol 7α‐hydroxylase
CYPs: Cytochrome P450s
DCA: Deoxycholic acid
DMEs: Drug metabolic enzymes
EC: Enterohepatic circulation
FXR: Farnesoid X receptor
G‐CA: Glyco‐cholic acid
G‐CDCA: Glyco‐chenodeoxycholic acid
G‐DCA: Glyco‐deoxycholic acid
GM: Gut microbiota
G‐UDCA: Glyco‐ursodeoxycholic acid
HCA: Hyodexycholic acid
K‐3‐G: Kaempferol‐3‐glucuronide
K‐7‐G: Kaempferol‐7‐glucuronide
K‐7‐S: Kaempferol‐7‐sulfate
Kae: Kaempferol
LCA: Lithocholic acid
SHP: Small heterodimer partner
SULTs: Sulfotransferases
T‐CA: Tauro‐cholic acid
T‐CDCA: Tauro‐chenodeoxycholic
T‐DCA: Tauro‐deoxycholic acid
T‐HDCA: Tauro‐hyodexycholic acid
T‐LCA: Tauro‐lithocholic acid
T‐UDCA: Tauro‐ursodeoxycholic acid
T‐αMCA: Tauro‐α‐muricholic acid
T‐βMCA: Tauro‐β‐muricholic acid
UDCA: Ursodeoxycholic acid
UGTs: UDP‐Glucuronosyltransferases
α‐MCA: α‐Muricholic acid
β‐MCA: β‐Muricholic acid
ω‐MCA: ω‐Muricholic acid

## 1 Introduction

An increasing body of research indicates a strong link between polyphenol consumption and the mitigation of metabolic syndrome (Wang et al. 2022). Kaempferol (Kae), a common natural polyphenol, is rich in a variety of plant‐derived products, in many dietary sources and traditional herbal medicines. These sources include an array of fruits and vegetables such as broccoli, tea, endive, cabbage, tomato, kale, leek, beans, grapes, and strawberries. Variability in dietary Kae intake is observed among individuals (Murphy, Wilkens, and Henderson 2007). But the daily intake of Kae into our bodies undergoes what kind of metabolism, and what effects does it have on our body? Our prior research has identified that Kae is predominantly metabolized by hepatic drug metabolic enzymes (DMEs), notably Cytochrome P450s (CYPs), Sulfotransferases (SULTs), and UDP‐Glucuronosyltransferases (UGTs). Among these, the conjugative reactions catalyzed by UGT and SULT enzymes constitute the predominant metabolic pathway, leading to the formation of Kae‐7‐glucuronide (K‐7G), Kae‐3‐glucuronide (K‐3G), and Kae‐7‐sulfate (K‐7S) (Zheng et al. 2016). In vivo, Kae undergoes a triple recycling process, including enterohepatic, local recycling, and enteric. This metabolic characteristic significantly enhances the Kae's local bioavailability, with UGT enzymes playing a crucial role in modulating these recycling mechanisms (Wang et al. 2019). When the triple recyclings of Kae were weakened, our previous results displayed that its anti‐inflammatory effect was also diminished (Liu et al. 2022). Given Kae's status as a dietary compound, a comprehensive investigation of its metabolic profile and its effects on metabolic syndrome is imperative.

Upon examining the expression of DMEs following long‐term Kae administration, we observed a notable trend: Kae suppressed the expression of CYP7A1 while enhancing the expression of CYP27A1 and UGTs. CYP7A1 and CYP27A1 are key DMEs that are essential for the metabolism of both xenobiotics and the synthesis of endogenous bile acids (BAs). Conversely, BAs, similar to Kae, are subject to enterohepatic circulation, predominantly governed by DMEs and gut microbiota (GM). The question arises: What are the effects of long‐term exogenous Kae consumption on its own metabolism and the synthesis of endogenous BAs?

Bile acids in hepatocytes are synthesized via two main biosynthetic pathways: the “classical” and “alternative” pathways. The classical pathway, predominantly driven by CYP7A1 and CYP27A1, produces the primary BAs, such as cholic acid (CA) and chenodeoxycholic acid (CDCA). The alternative pathway, mainly catalyzed by CYP27A1, leads to CDCA production. Both CDCA and CA are converted into conjugated BAs (CBA) through the action of various synthetase enzymes, including bile acyl‐CoA synthetase (BACS), bile acid‐CoA:amino acid N‐acyltransferase (BAAT), Cysteine dioxygenase type 1 (CDO1), Cysteine sulfinic acid decarboxylase (CSAD), the Sodium‐ and chloride‐dependent taurine transporter (TAUT), and UGTs. In the liver, BAs could serve as the natural ligands for the Farnesoid X receptor (FXR), acting as either potent agonists to activate or antagonists to inhibit its function (Wang et al. 2021). Conversely, FXR also modulates the expression of various target genes, including regulating the expressions of CYPs and UGTs (Jarrar and Lee 2021; Deng et al. 2024). Current research confirms that the FXR‐small heterodimer partner (Shp) and FXR‐fibroblast growth factor 15 (Fgf15) pathways are key regulators of BA synthesis and metabolism (Guan et al. 2022). The hepatic FXR‐Shp signaling axis is instrumental in maintaining BA homeostasis by orchestrating the synthesis, metabolism, and circulation of BAs (Ocvirk and O'Keefe 2017). On the other hand, intestinal‐derived Fgf15 reaches the liver via portal vein circulation and activates the receptor complex of Fgf receptor 4 (Fgfr4)‐β‐klotho (Klβ), inhibiting the CYP7A1 expression (Katafuchi and Makishima 2022). Concurrently, BAs synthesized in hepatocytes are secreted into the intestinal lumen, where they undergo further metabolism by GM (De Aguiar Vallim, Tarling, and Edwards 2013; Lazar et al. 2019). This process underscores the GM's crucial role in determining BA composition and maintaining BA homeostasis. For instance, primary BAs such as CA and CDCA can undergo 7α‐dehydroxylation by GM, leading to the formation of secondary BAs, including deoxycholic acid (DCA) and lithocholic acid (LCA), a process linked to the metabolic activities of Clostridium and Eubacterium species. Additionally, CBAs are converted into unconjugated BAs (UBAs) through the action of bile salt hydrolases (BSH) secreted by specific GM members, including Bacteroides, Lactobacillus, Clostridium, and Listeria (Thomas et al. 2008; Jia, Xie, and Jia 2017). Exogenous chemicals that disrupt the GM can cause imbalances in BA homeostasis. The complex interplay and regulatory mechanisms between BAs and GM have been linked to a variety of metabolic diseases (Cai et al. 2022a; Cai, Sun, and Gonzalez 2022b). These include, but are not limited to, type 2 diabetes, obesity, nonalcoholic fatty liver disease, and inflammatory bowel disease. In summary, CYPs and GM jointly regulate the homeostasis of BAs.

An increasing number of studies highlight the crucial role of the GM in enhancing the bioavailability and functionality of dietary phytochemicals, particularly polyphenolic compounds (Guang et al. 2014; Zimmermann et al. 2019). Furthermore, evidence is mounting to suggest that polyphenols possess prebiotic properties, which can ameliorate metabolic disorders such as obesity, hyperlipidemia, and diabetes (Sandoval et al. 2020; Kasprzak‐drozd et al. 2021). Specifically, prebiotics can slow disease progression by promoting the growth of beneficial bacteria and simultaneously inhibiting the growth of harmful bacterial strains (Bindels et al. 2015). Here, we treated the mice with Kae for 15 days and compared the pharmacokinetics of Kae at day 0 and day 15 in different dosages. Moreover, LC–MS/MS and 16s rRNA amplicon sequencing were used to evaluate the BA pool and GM composition in the mice treated with Kae. Molecular docking and Pearson's correlation were also utilized to delineate the relationship between the changed GM composition and the BAs metabolism.

## 2 Materials and Methods

### 2.1 Chemicals Reagents

Chenodeoxycholic acid, CA, DCA, LCA, Ursodeoxycholic acid (UDCA), Tauro‐chenodeoxycholic acid (T‐CDCA), Glyco‐chenodeoxycholic acid (G‐CDCA), Tauro‐cholic acid (T‐CA), Glyco‐cholic acid (G‐CA), Tauro‐ deoxycholic acid (T‐DCA), Tauro‐lithocholic acid (T‐LCA), Tauro‐β‐muricholic acid (T‐βMCA), Tauro‐ursodeoxycholic acid (T‐UDCA), and Glyco‐deoxycholic acid (G‐DCA) were purchased from Dalian Meilun Biotechnology Co. LTD. Hyocholic acid (HCA) was purchased from ChemCruzTM Biochemical company. Hyodexycholic acid (HDCA) and the internal standard (IS, DCA‐d5) were purchased from Sigma‐Aldrich (St Louis, MO). α‐Muricholic acid (αMCA), β‐muricholic acid (βMCA), ω‐muricholic acid (ωMCA), Tauro‐α‐muricholic acid (T‐αMCA), Tauro‐hyodexycholic acid (T‐HDCA), and Glyco‐ursodeoxycholic acid (G‐UDCA) were purchased from Toronto Research Chemicals Inc. (TRC). Kae and K‐3‐G were purchased from Chengdu Must Bio‐Technology Co. Ltd. and Extrasynthese (Lyon, Rhône, French), respectively. K‐7‐G and K‐7‐S were obtained from Karebay Biochem (Shanghai, China). Phenylmethanesulfonyl fluoride and dithiothreitol were bought from Sigma‐Aldrich Co. (St. Louis, MO, USA). The sequencing grade modified trypsin was obtained from Promega (Madison, WI). The peptides of DMEs and internal standard (IS, purity > 95%) were purchased from Your R&D Partner. HPLC‐grade formic acid, acetonitrile, and methanol were acquired from Merck (Darmstadt, Germany). Coomassie brilliant blue for protein measurement was purchased from Bio‐Rad (Hercules, California, USA).

### 2.2 Mouse Treatment

Male C57BL/6 mice were purchased from the Experimental Animal Center of Sun Yat‐sen University. All animal experiments were conducted in strict accordance with the guidelines approved by the Institutional Animal Care and Use Committee of the International Institute for Translational Chinese Medicine (IITCM_20180140). The animals were housed in a controlled environment with a relative humidity of 50%–60% and an ambient temperature of 24°C–26°C, under a regulated 12‐h light–dark cycle to ensure their well‐being and the validity of the experimental conditions. Upon completion of the experimental procedures, the animals were subjected to a 12‐h fasting period, during which they continued to have unlimited access to water. The mice were euthanized by cervical dislocation after anesthesia via inhaling isoflurane.

### 2.3 Experimental Design

Male C57BL/6 mice, aged 6 weeks, were randomly assigned to three experimental groups. The first group served as the control, receiving a diet supplemented with 2.5% Na_2_CO_3_ (Ctr, *n* = 8). The other two groups were treated with Kae at a dosage of 50 mg/kg (K50, *n* = 8) and 100 mg/kg (K100, *n* = 8), respectively. Firstly (0‐day), blood samples (approximately 15 μL) were collected through snipping the mouse tail near its tip at 3, 6, 12, 20, 30, 60, 120, 180, 240, 360, 540, and 660 min after K50 and K100 treatment, respectively. Then continuous gavage was implemented daily for 2 weeks. When the second blood samples were collected (15‐day), the time without Kae treatment was more than 48 h since the last Kae administration. The plasma was separated from blood sample by centrifuging at 8000 rpm for 5 min. Feces were collected at 15‐day. Liver, plasma, gallbladder, and fecal samples were secured in a state of deep freeze at −80°C, immediately after sacrifice.

### 2.4 Pharmacokinetic Studies

The separated plasma (5 μL) and genistein (5 μL internal standard) were mixed, and 190 μL methanol was added. The mixture was swirled and centrifuged at 20,817 g for 30 min at 4°C. 160 μL supernatant was taken and dried in a nitrogen dryer, then redissolved with 50% methanol (100 μL) and centrifuged for LC–MS/MS analysis. The detailed experimental procedure of LC–MS/MS analysis was in accordance with our previously described method (Zheng et al. 2016; Liu et al. 2022).

### 2.5 BA Analysis via LC–MS/MS Method

The plasma, gallbladder, liver, and fecal samples were meticulously prepared for the quantification of BAs. We analyzed a comprehensive panel of 12 CBAs, encompassing T‐CA, G‐CA, T‐CDCA, G‐CDCA, T‐βMCA, T‐αMCA, T‐DCA, G‐DCA, T‐UDCA, G‐UDCA, T‐HDCA, T‐LCA, and 10 UBAs, including LCA, HDCA, ωMCA, CA, βMCA, HCA, αMCA, CDCA, UDCA, and DCA. These BAs were detected using a sensitive LC–MS/MS approach. The concentrations of BAs were measured in the gallbladder, plasma, feces, liver, and intestinal contents. The sample processing techniques and analytical conditions were meticulously aligned with the well‐established methods previously reported in the literature (Li et al. 2022).

### 2.6 LC–MS/MS Analysis of DMEs

Mouse tissues (liver, intestine, colon, and kidney) were isolated, shredded, and washed with cold saline, respectively. A cold buffer saline solution with 0.28 mM phenylmethyl sulfonyl fluoride (PMSF) as a protease inhibitor was gently mixed with the minced tissues to facilitate homogenization. Then, the resulting suspension was subjected to centrifugation at a high speed of 9000 g for 20 min at 4°C. The suspension was collected and further hydrolysis to polypeptide for LC–MS/MS analysis. The meticulous processing methodologies and stringent testing conditions employed in this study were in strict accordance with those detailed in our previous research (Chen et al. 2017, Li et al. 2020).

### 2.7 Molecular Docking

The protein structure of human FXR (6HL1) was retrieved from the Protein Data Bank (PDB) (https://www.rcsb.org/). For the chemical structure of Kae (PubChem ID: 5280863), we got its three‐dimensional structure from PubChem (https://pubchem.ncbi.nlm.nih.gov/). We used AutoDockTools to optimize the receptor protein for docking, adding hydrogen atoms, and balancing charges. AutoDock Vina 1.1.2 software (Trott and Olson 2010) was used to perform molecular docking of the receptor protein (6HL1) and small ligand (Kae) molecule. The complex of 6HL1 and endogenous agonist CDCA were further analyzed through Protein Plus debase (https://proteins.plus/).

### 2.8 The Assay of BSH Activity

Fecal samples (50 mg/mouse) were mixed with 500 μL of cold phosphate‐buffered saline solution containing 1 mM PMSF using a motorized homogenizer. The mixture was then sonicated in an ice bath for 30 min and centrifuged at 12,000 g for 15 min at 4°C. Protein was obtained from the supernatant and the protein concentration was determined using the BCA protein assay kit (Bio‐Rad, Beyotime, China). For the incubation process, the samples were incubated in a 37°C environment with a 3 mM sodium acetate buffer at pH 5.2, along with 0.1 μg of fecal protein and varying concentrations of the substrate T‐CDCA‐d5 (1 μM, 5 μM, and 10 μM). After a 30‐min incubation period, 100 μL of the internal standard (LCA‐d4) was added to the reaction mixture. Following centrifugation, the supernatant was carefully collected and prepared for LC–MS/MS analysis.

### 2.9 Quantitative Reverse Transcription Polymerase Chain Reaction (qRT‐PCR)

The qRT‐PCR was conducted according to our previous study (Li et al. 2022). Seventeen genes were selected as target detectors: *Cyp7a1*, *Cyp27a1*, *Fxr*, *Shp*, *Fgr15*, *Baat*, *Bacs*, *Cdo1*, *Csad*, and *Taut*. A house keeping gene, *Gapdh*, was applied as the internal control. The levels of gene expression were determined by qPCR using the Power SYBR Green PCR master mix (Applied Biosystems, CA, USA) in QuantStudio5 system (Applied Biosystems). Primer sequences are detailed in Table S1. mRNA expression levels were standardized to *Gapdh* for consistency, with data analyzed using the 2^−ΔΔCt^ formula.

### 2.10 Western Blot

Mouse liver samples were collected and two samples per group were combined for protein analysis. Three replicates of these pooled samples were maintained for each experimental set. Total protein was isolated using RIPA buffer from Sigma‐Aldrich. The proteins were then examined with antibodies specific to β‐ACTIN, CYP27A1, CYP7A1, and FXR (all Santa Cruz, diluted 1:1000). Detection was facilitated by secondary antibodies against mouse and rabbit IgG (SAB, diluted accordingly). The methodology followed was in line with our previous reports, ensuring consistency in processing (Li et al. 2022).

### 2.11 Fecal DNA Extraction and ERIC‐PCR Analysis

Total genomic DNA was extracted from fecal samples using QIAamp DNA Stool Mini Kit (QIAGEN) according to the manufacturer's protocol. The extracted DNA samples were analyzed for the similarity of GM composition using ERIC‐PCR as described (Huang et al. 2017). The resulting DNA banding patterns on the gel were digitized by Image Lab 3.0 system (Bio‐Rad). Based on the distance and the intensity of each DNA bands, SIMCA‐P 14.0 tool (Umetrics, Umea, Sweden) with 95% (*p* < 0.05) confidence level was applied to obtain the PLS‐DA score plots (Huang et al. 2017).

### 2.12 16S rRNA Amplicon Sequencing

The methods used for processing were in accordance with our established protocols (Li et al. 2022).

### 2.13 Data Analysis

Statistical differences were assessed using one‐way ANOVA for normally distributed data and Kruskal‐Wallis H for non‐normally distributed data, both conducted in SPSS 19.0. Alpha and beta diversity analysis and partial least squares discriminant analysis (PLS‐DA) were analyzed using R and SIMCA‐P 14.0 tool, respectively. Correlation analyses were performed using SPSS 19.0, according to the Pearson product–moment correlation for normal related data and Spearman's rank correlation for non‐normally related data. Significance levels were set at *p* < 0.05 (*), *p* < 0.01 (**) and *p* < 0.001 (***).

## 3 Results

### 3.1 Pharmacokinetic Characteristics of Kae, K‐7G, K‐3G, and K‐7S in C57BL/6 Mice

**FIGURE 1:**
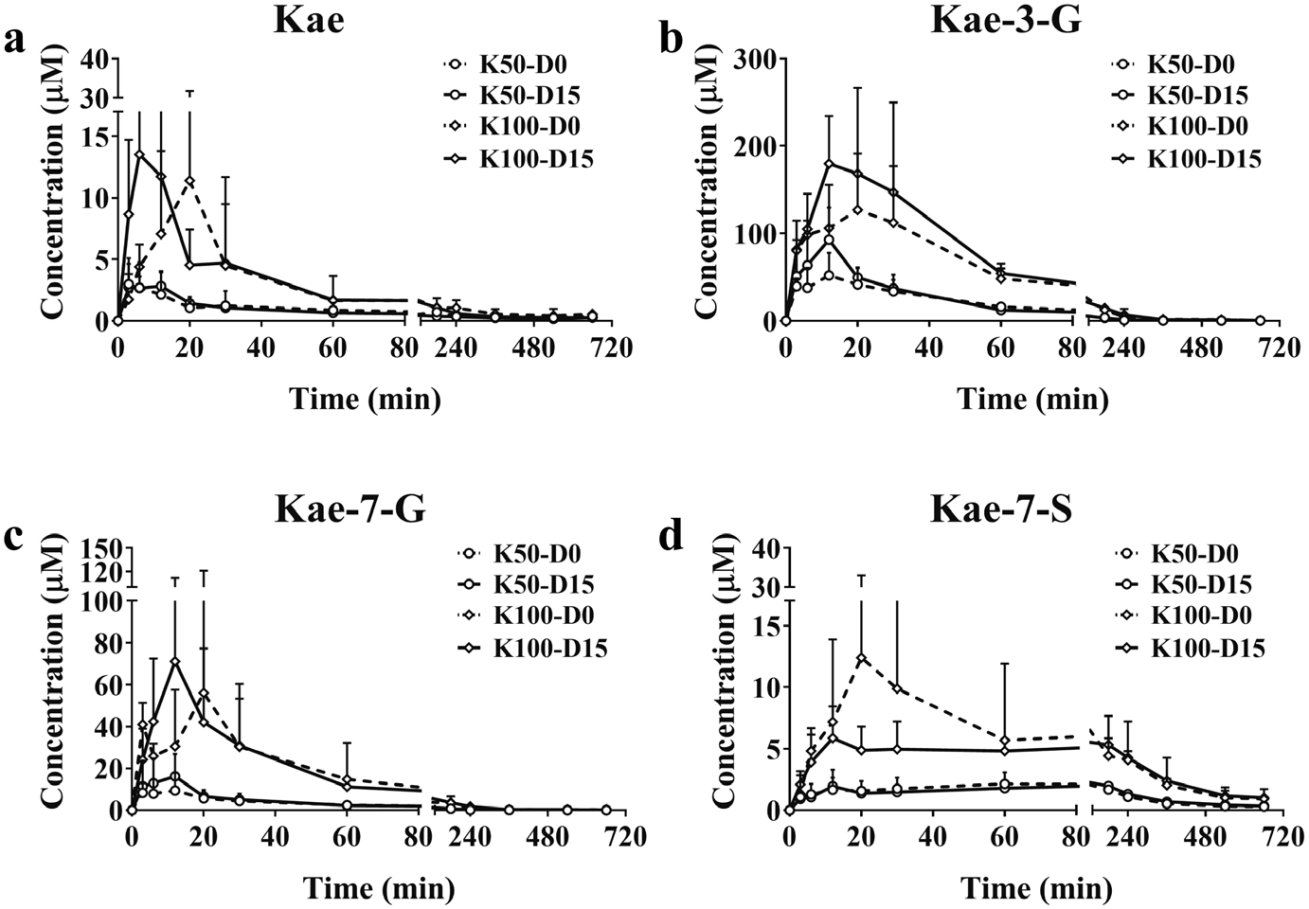
Concentration‐time curves of plasma concentrations of Kae and its metabolites. Mean plasma concentrations of Kae (a), K‐3‐G (b), K‐7‐G (c), and K‐7‐S (d) in C57BL/6 mice after an oral administration of Kae (50 and 100 mg/kg). Data represent mean ± SD (*n* = 4).

15‐day Kae treatment showed no significant differences in food intake, water consumption, and body weight among the Ctr, K50, and K100 groups (Figure S1a). Additionally, none of the treated mice showed variations in the organ index (Figure S1b). At 0‐day and 15‐day, the concentrations of Kae, K‐7G, K‐3G, and K‐7S were determined and their mean plasma concentration‐time curves are displayed in Figure 1, respectively. The corresponding pharmacokinetic parameters are shown in Table S2. Once orally administered, Kae was quickly absorbed and metabolized into K‐7G, K‐3G, and K‐7S. For Kae, from the parameters of the time to peak drug concentration (T_max_) and the maximum concentration (C_max_), it can be inferred that long‐term intervention with Kae can accelerate the absorption of Kae in body; for K‐3G, C_max_ was 1.9‐fold higher than the 0‐day groups, *p* = 0.07; for K‐7G, C_max_ was increased compared with the 0‐day groups (1.9‐ and 1.3‐fold, *p* = 0.23 and 0.51, respectively); for K‐7S, no significant changes were observed. For K‐3G and K‐7G, the area under the plasma concentration‐time curve from 0 to last time (AUC_0–t_) and the area under the plasma concentration‐time curve from 0 to infinite time (AUC_0–∞_) display similar increased trend after Kae treatment for 15 days (1.1~1.8 folds).

### 3.2 Effect of Kae on the Expression of DMEs in Liver Tissue

**FIGURE 2:**
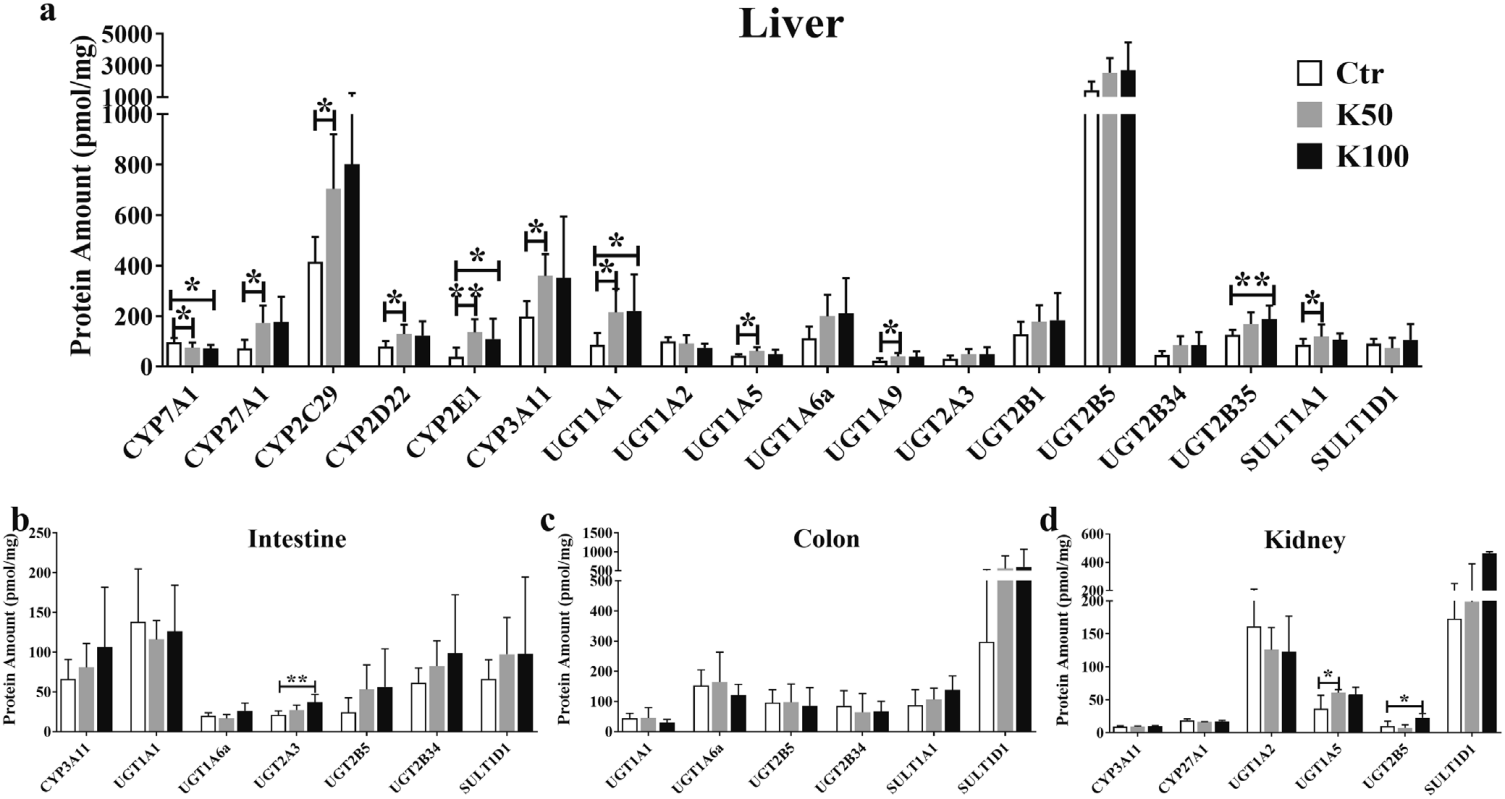
Effect of Kae on liver DMEs in C57BL/6 mice. The protein amounts of CYPs, UGTs, and SULTs in the liver (a), intestine (b), colon (c), and kidney (d) tissue were quantified using the established LC–MS/MS method. Data are depicted as mean ± SD (*n* = 7). Statistical significance is indicated by * for *p* < 0.05 and ** for *p* < 0.01 across all analyses. Ctr for untreated C57BL/6 mice; K50 for C57BL/6 mice given 50 mg/kg Kae; K100 for C57BL/6 mice given 100 mg/kg Kae.

After 15‐day Kae treatment with a dosage of K50 and K100, we observed the up‐regulation of protein amounts in liver tissue (Figure 2a), including CYP2C29 (1.7‐ and 1.9‐fold), 2D22 (1.6‐fold), 2E1 (3.5‐ and 2.8‐fold), and 3A11 (1.8‐fold), compared with the control group; the UGT isoforms were observed higher expression, including UGT1A1 (2.5‐ and 2.6‐fold), 1A6a (1.8‐ and 1.9‐fold), 1A9 (1.8‐ and 1.7‐fold), 2A3 (1.7‐ and 1.6‐fold), 2B5 (1.8‐ and 1.9‐fold), and 2B34 (1.8‐ and 1.9‐fold); there was no significant change in SULT1A1 and SULT1D1. Among them, the amounts of CYP7A1 and CYP27A1 display an opposite result, an increased and a decreased expression after K50 and K100 treatment (1.3~1.7‐fold, *p* < 0.05). There are no significant changes in intestine, colon, and kidney tissue (except for UGT2A3 in intestine, UGT1A5 and 2B5 in kidney tissue, Figure 2b–d).

### 3.3 Effect of Kae on the Expression Levels of CYP7A1 and CYP27A1

**FIGURE 3:**
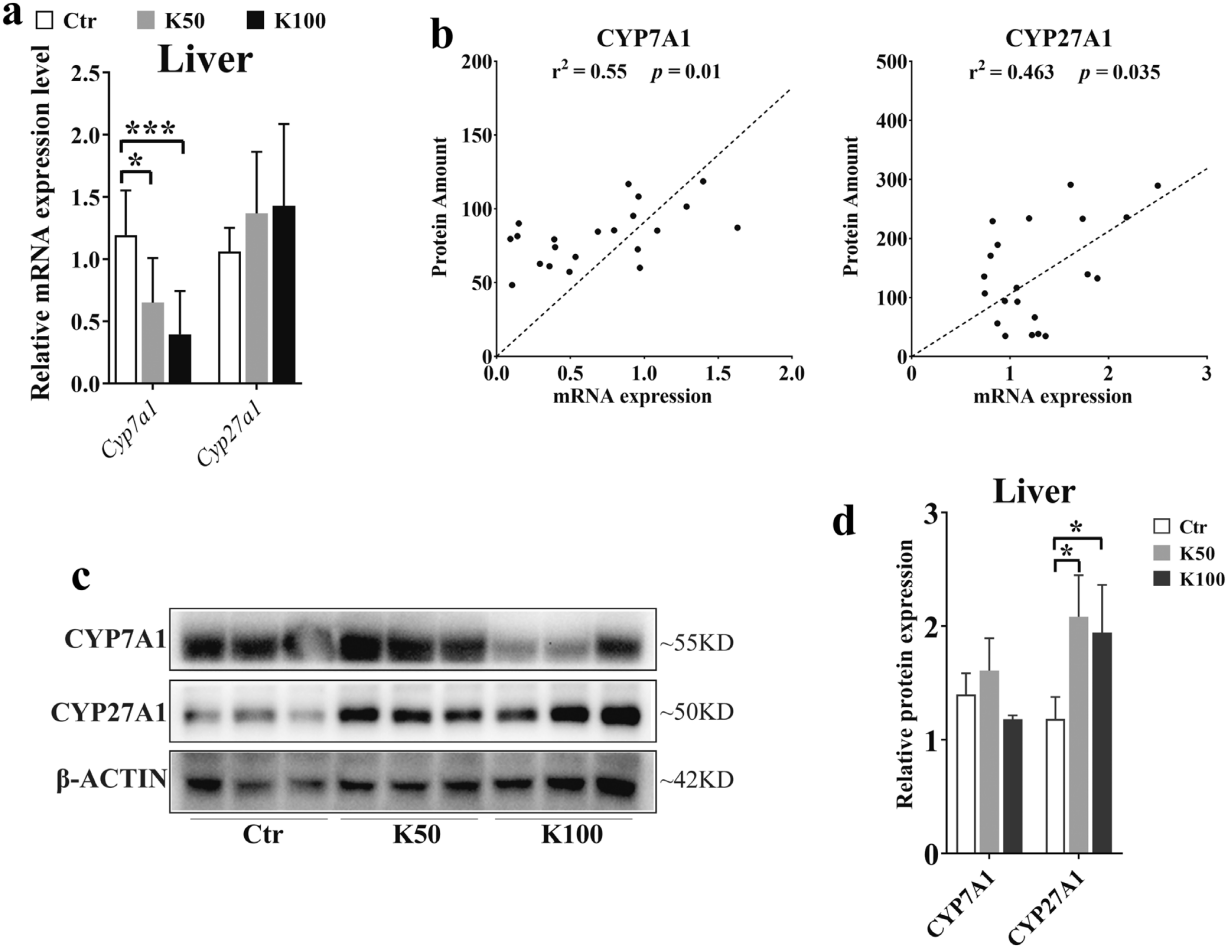
Effects of Kae on the expression of CYP7A1 and CYP27A1 in the liver tissues of the Ctr, K50, and K100 groups. (a) mRNA expression levels detected by qRT‐PCR method. (b) The correlations between mRNA (detected by qRT‐PCR method) and protein expressions (detected by LC–MS/MS method) of CYP7A1 and CYP27A1. (c, d) Protein expressions detected by western blotting. Data are depicted as mean ± SD (*n* = 7). Statistical significance is indicated by * for *p* < 0.05 and *** for *p* < 0.001 across all analyses.

Based on the results from Figure 2, we further confirmed the expression of CYP7A1 and CYP27A1. Figure 3a demonstrates that the *Cyp7a1* mRNA levels were downregulated by 1.7‐fold in the K50 group and 2.8‐fold in the K100 group compared to the Ctr, both with statistical significance (*p* < 0.05). And the mRNA expression of *Cyp27a1* was increased by 1.7‐ and 1.4‐fold in Kae‐treated groups (*p* > 0.05). We further analyzed the relationship between mRNA expression and protein amounts of CYP7A1 and CYP27A1, and we found that there was a closed relationship between the mRNA expression and the protein amounts (*r* = 0.55, *p* = 0.01 and *r* = 0.463, *p* = 0.035, Figure 3b). Moreover, the western blotting results also showed that the CYP7A1 was downregulated in K100 group. Whereas the expression of CYP27A1 was upregulated in both K50 and K100 groups compared to the Ctr group (Figure 3c,d).

### 3.4 Effect of Kae on BAs Pool in C57BL/6 Mice

**FIGURE 4:**
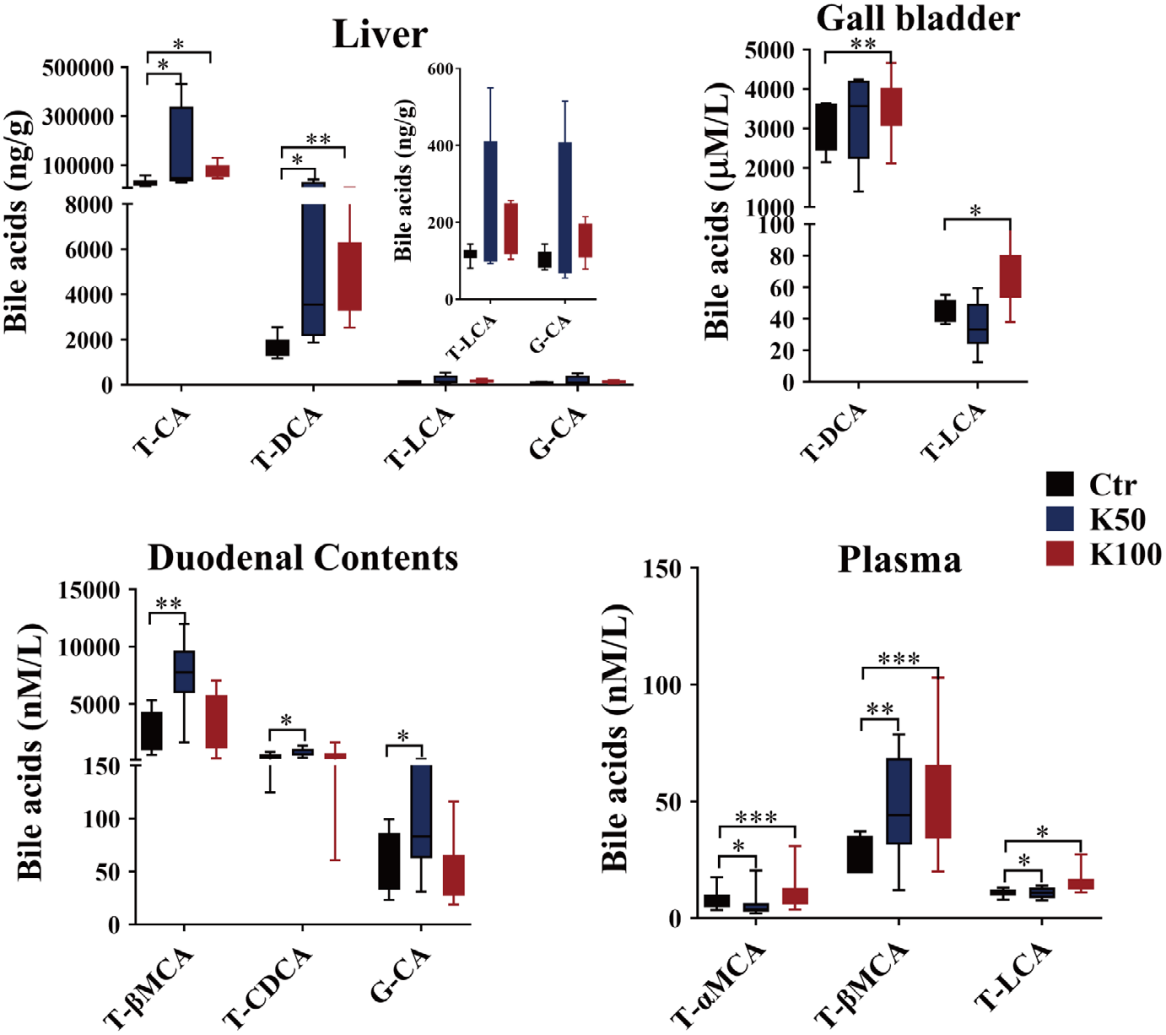
Effects of Kae on the BA pool in C57BL/6 mice. The BA levels were assessed in the liver, gallbladder, duodenal contents, and plasma across the Ctr, K50, and K100 groups. Data are depicted as mean ± SD (*n* = 8), *p* < 0.05 (*), *p* < 0.01 (**), and *p* < 0.001 (***). Duo, duodenal contents.

There were 12 CBAs and 10 UBAs, which were detected using LC–MS/MS method (Figure 4 and Figure S2). In liver tissues, T‐CA and T‐DCA markedly rose by 2.5 to 7.5‐fold in the K50 and K100 groups versus the Ctr (*p* < 0.05); in the gallbladder, the amounts of T‐DCA and T‐LCA were observably upregulated in K100 group than in the Ctr group (1.4 folds, *p* < 0.05); in duodenal contents, the K50 group exhibited significant upregulation of T‐βMCA, T‐CDCA, and G‐CA by 2.8‐, 1.9‐ and 1.7‐fold respectively compared to the Ctr group (*p* < 0.05); in plasma, the levels of T‐αMCA, T‐βMCA, and T‐LCA were significantly upregulated in the Kae treatment groups compared with the Ctr group (1.4–37.5 folds, *p* < 0.05).

### 3.5 Kae Activates the Liver FXR Expression and Reduced the BSH Activity

**FIGURE 5:**
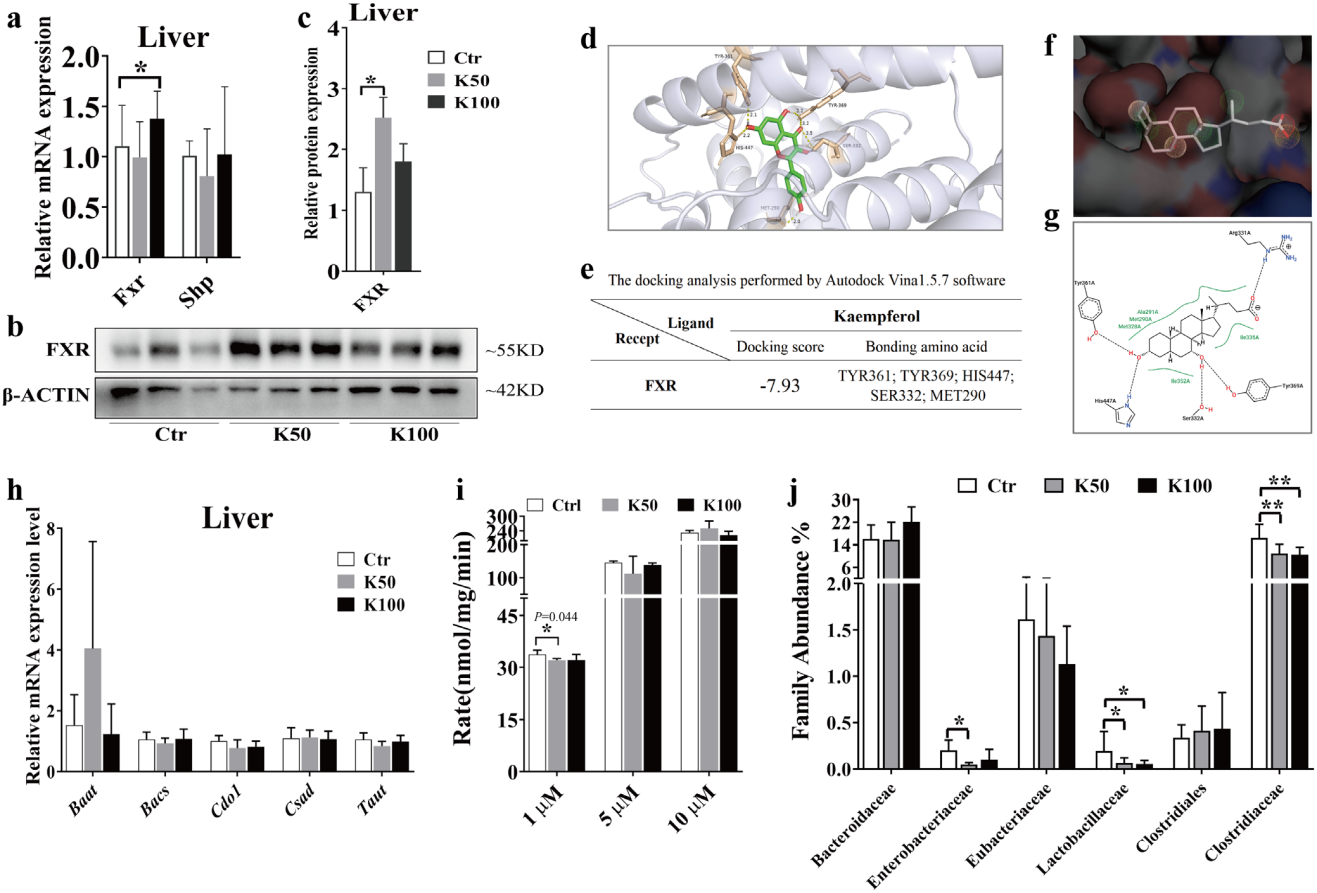
Effect of Kae on FXR, enzymes synthesizing conjugated BAs, and the BSH activity. (a) The mRNA expression levels of *Fxr* and *Shp*. (b, c) The FXR protein expression. (d, e) The molecular docking results between Kae and human FXR; (f, g) the binding amino acid residues between human FXR and endogenous agonist CDCA; (h) the mRNA expressions of some enzymes synthesizing conjugated BAs. (i) The BSH activity measured by the hydrolysis rate of substrate T‐CDCA‐d5. (j) The abundance of family that can secrete BSH hydrolase. Data are depicted as mean ± SD (*n* = 7). Statistical significance is indicated by * for *p* < 0.05 and ** for *p* < 0.01 across all analyses.

The *Fxr* mRNA level was significantly increased in the K100 group compared with the Ctr group (1.4‐fold, *p* < 0.05, Figure 5a). *Shp* and *Fgf15* display an increasing tendency without statistical significance. Especially for the *Fgf15*, there is significant individual variation among the groups (Figure S3). Western blotting results showed that the expression of FXR was significantly increased in the K50 and K100 groups when compared to the Ctr group (1.9‐ and 1.4‐fold, respectively, Figure 5b,c). The molecular docking results showed that the predicted binding sites between Kae and human FXR with the amino acid residues TYR361, TYR369, HIS447, SER332 by hydrogen bonding, and MET290 through hydrophobic interactions (Figure 5d). The docking score reaches −7.93 kcal/J (Figure 5e). Then we further analyzed the complex of 6HL1 and endogenous agonist CDCA through Protein Plus debase and found that the main pharmacodynamic groups of the agonist CDCA are similar to Kae's. Moreover, the activation site (the binding amino acid residues) of CDCA on the protein is identical to that of Kae (Figure 5f,g). Figure S4 also indicates that FXR (gene name Nr1h4) acts as an important regulatory molecule in the process of Kae regulating BA metabolism.

As shown in Figure 5h, there are no significant differences in liver enzymes responsible for the synthesis of conjugated BA following Kae treatment. While, Kae slightly reduced the BSH activity in mouse feces after Kae treatment, especially in the low‐dose group (*p* = 0.044, Figure 5i). Consequently, we further investigated the abundance of bacteria capable of secreting BSH hydrolases. As shown in Figure 5j, Lactobacillaceae significantly decreased in the K50 and K100 groups when compared to the Ctr group (3.1‐ and 3.7‐fold, respectively, *p* < 0.05); Clostridiaceae decreased in the K50 and K100 groups than in the Ctr group (1.5 and 1.6 folds, respectively, *p* < 0.05); Enterobacteriaceae also showed a decrease in the Kae treatment groups compared with the Ctr group (4.1 and 2.1 folds, respectively, *p* < 0.05).

### 3.6 The Change of GM Composition and the Association With Differential BA

**FIGURE 6:**
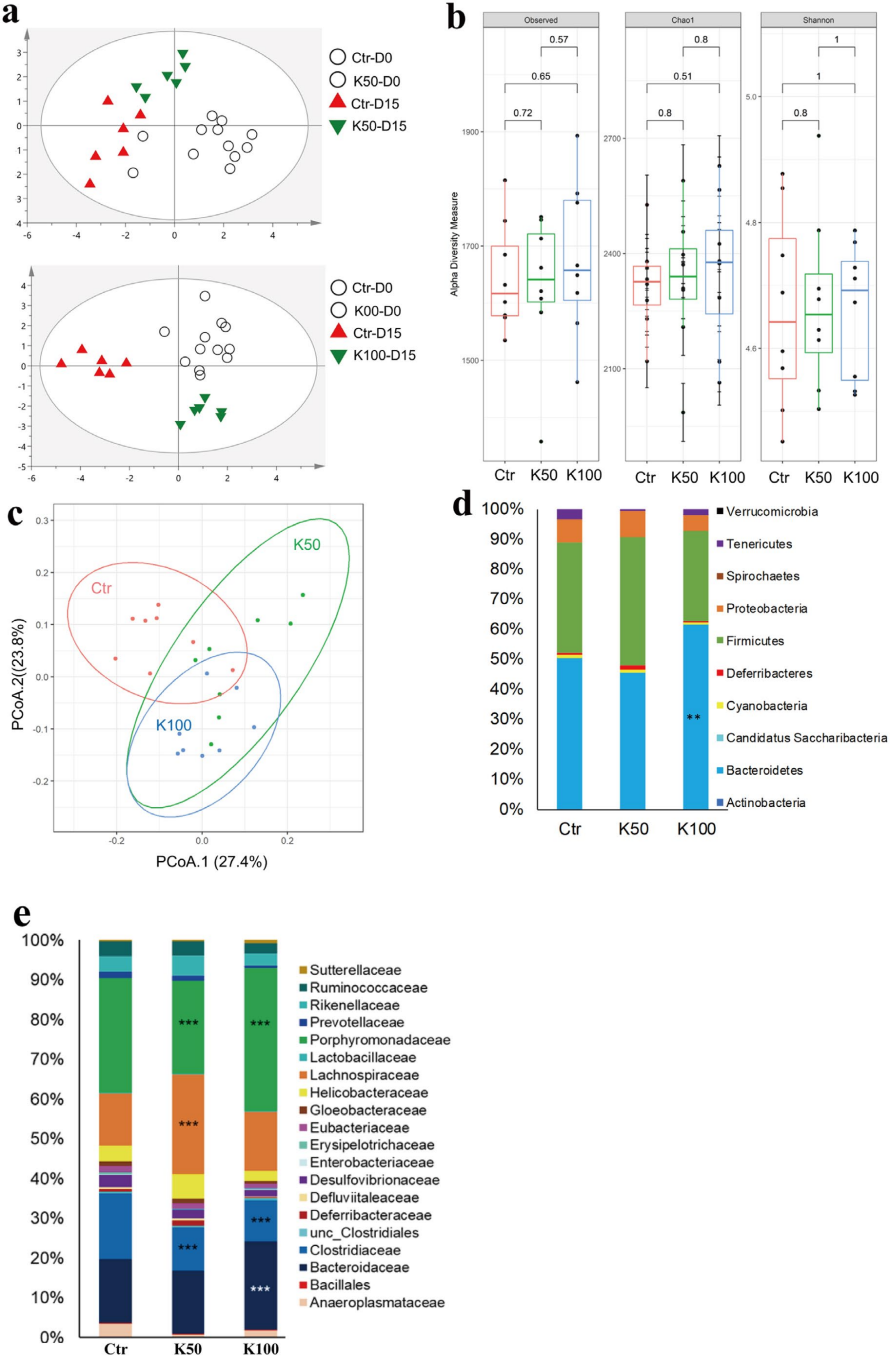
Effect of Kae on GM composition in C57BL/6 mice. (a) PLS‐DA plots of ERIC‐PCR DNA profiles of mice in the Ctr, K50, and K100 groups. Each symbol in the PLS‐DA plots represents the GM profile of an individual mouse. (b) Alpha diversity analysis of total operational taxonomic units (OTUs) was analyzed from 16S rRNA gene sequencing data, with significance determined by the Kruskal‐Wallis test. (c) Weighted UniFrac distance analysis of the total OTUs in the fecal DNA of Ctr, K50, and K100 group mice. (d) Relative percentage abundances of phyla. The y‐axis shows the percentage abundances of the phyla. (e) Dominantly detected families. These families account for > 99.5% of each group. This list includes only families present in > 50% of the samples in each group. The y‐axis shows the percentage abundances of the families. Statistics were calculated with the Kruskal‐Wallis test. (**p < 0.01, ***p < 0.001; control versus treatment, *n* = 8).

**FIGURE 7:**
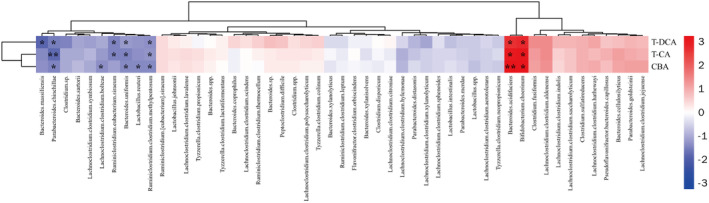
The correlation between the changed BAs and the BSH‐possessing GM. Heatmap was presented as the Spearman's correlation among the changed BA and the GM possessing the BSH activity. *p ≤ 0.05, **p ≤ 0.01.

In this study, we first investigated the GM profile of the fecal DNA samples from experimental mice using the ERIC‐PCR technique. The PCA plot showed a clear separation among the K50‐, K100‐, and the control groups (Figure 6a). To further analyze the alteration of GM composition after Kae treatment, 16S amplicon sequencing was performed. Alpha diversity analysis showed that K50 and K100 increased the observed, Chao 1, and Shannon index compared to the control group (Figure 6b). Furthermore, weighted UniFrac distance analysis showed a difference between the mice treated with Kae and control mice (Figure 6c). At the phylum level, K50 enhanced Firmicutes' prevalence while reducing Tenericutes. K100 significantly increases the relative abundance of Bacteroidetes but decreases Tenericutes (Figure 6d). Moreover, the difference also existed among the groups in the family taxon (Figure 6e). The Bacteroidaceae, Lachnospiraceae, and Porphyromonadaceae were significantly increased, while the Clostridiaceae markedly decreased after Kae treatment. The previous reports showed that changes in GM composition, including Bacteroides, Clostridium, Lactobacillus, Bifidobacterium, and Listeris, were correlated to the de‐conjugation of BSH (Jia, Xie, and Jia 2017). Here, our results (Figure 7) also showed that the metabolic products of BA, i.e., T‐CA, T‐DCA, and the total CBA in liver tissue were negatively related to the species of *Bacteroides uniformis* ( *B. uniformis* ), *Parabacteroides chinchilla* (*P. chinchilla*), *Ruminiclostridium clostridium methylpentosum* (*R. clostridium methylpentosum*), *Ruminiclostridium eubacterium siraeum* (*R. eubacterium siraeum*), *Lactobacillus reuteri* ( *L. reuteri* ), *Bacteroides sartorii* ( *B. sartorii* ), *Bacteroides massiliensis* ( *B. massiliensis* ), *Clostridium* sp., and *Lachnoclostridium clostridium bolteae* (*L. clostridium bolteae*) (*p* < 0.05). However, the concentration of T‐CA, T‐DCA, and the total CBA were positively related to the species of *Bacteroides acidifaciens* ( *B. acidifaciens* ) and *Bifidobacterium choerinum* ( *B. choerinum* ) (*p* < 0.05).

### 3.7 Kae Elevated the Beneficial GM in C57BL/6 Mice

**FIGURE 8:**
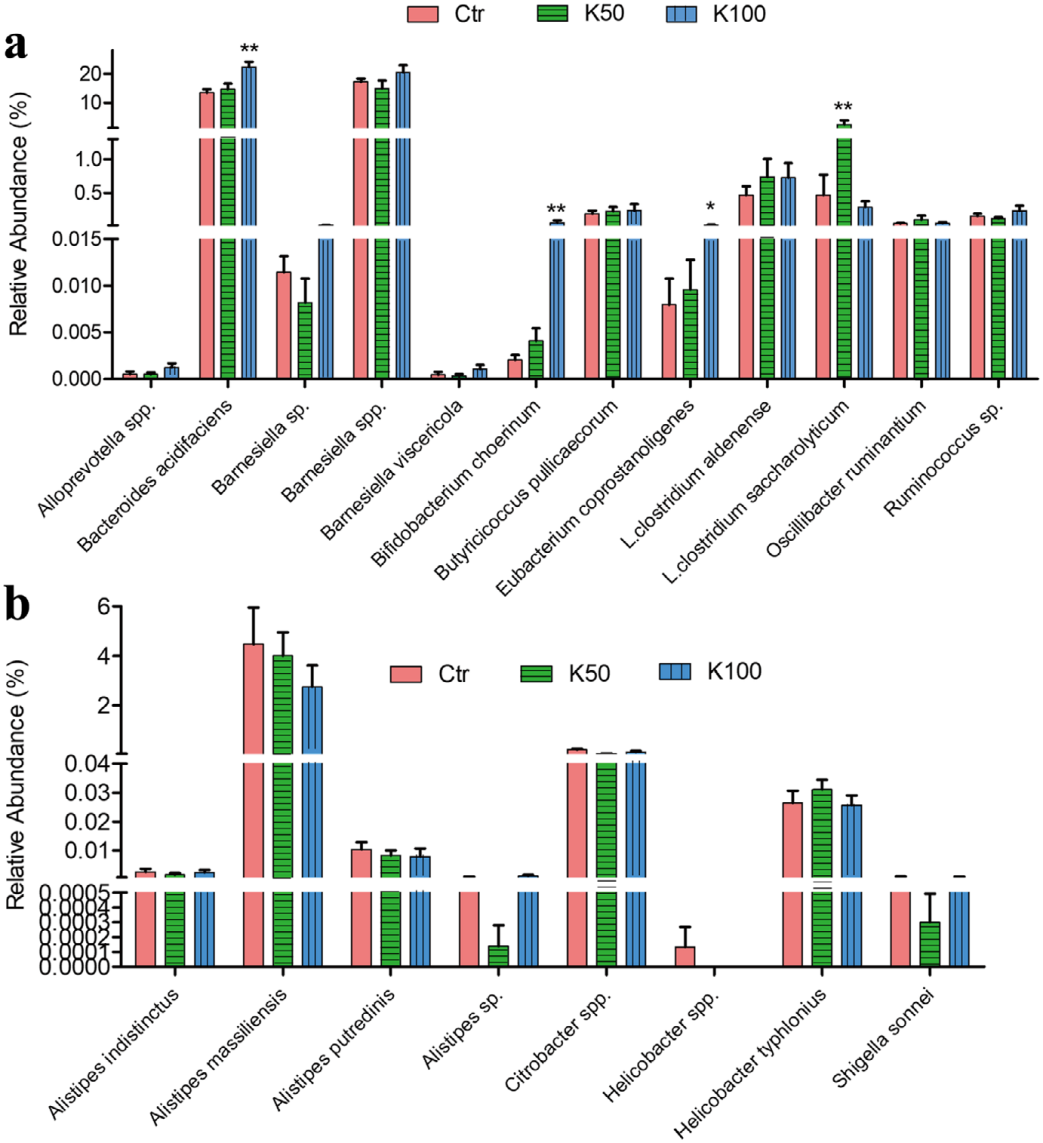
Kae enhanced the beneficial GM and suppressed the pathogenic GM in C57BL/6 mice. (a) List of potentially beneficial bacteria that were enriched in C57BL/6 mice following K50 or K100 treatment. (b) List of potential pathogens suppressed by Kae treatment. Statistics were calculated with the Kruskal‐Wallis test. (*p < 0.05, **p < 0.01; control versus treatment, *n* = 8). L., *Lachnoclostridium*.

Figure 8 and Figure S4 indicate that Kae can act as a prebiotic. Notably, several beneficial bacteria, including *B. acidifaciens* , *B. choerinum* , *Eubacterium coprostanoligenes* ( *E. coprostanoligenes* ), and *Lachnoclostridium saccharolyticum* (*L. saccharolyticum*) were increased in the mice treated with Kae (Figure 8a). Comparatively, K100 effectively suppressed the growth of certain pathogenic bacteria such as *Alistipes*, *Citrobacter* spp., and *Helicobacter* spp. (Figure 8b). Additionally, we further conducted a correlation analysis between these bacteria and key liver proteins related to BA synthesis and metabolism. The results (Figure S5) indicated that Cyp7a1 showed a negative correlation with probiotic *Butyricicoccus pullicaecorum* ( *B. pullicaecorum* ) and a positive correlation with harmful bacterium *Alistipes putredinis* ( *A. putredinis* ); FXR was significantly negatively correlated with harmful bacterium *Alistipes massiliensis* ( *A. massiliensis* ); Bacs and Taut exhibited significant positive correlations with some probiotics, such as *B. acidifaciens* and *Bamesiella viscericola* ( *B. viscericola* ).

## 4 Discussion

In our investigation, we observed that Kae did not exert any significant influence on parameters such as food intake, water consumption, body weight, and organ index in the subjects. However, we did note that Kae enhances its own absorption and metabolic circulation in vivo. Following 15‐day treatment of Kae, there were marked increases in the AUC_0–t/∞_ and C_max_ of Kae's glucuronide metabolites. Concurrently, T_max_ of Kae showed a tendency to decline, while its C_max_ exhibited an upward trend. This enhancement indicates a rise in absorption and systemic circulation, as depicted in Figure 1 and detailed in Table S2. Then we further studied the expression of DMEs and found the statistical changes of DMEs amounts after Kae treatment, especially the UGT enzymes. It is unsurprising that the expression of specific UGT isoforms was notably induced, particularly UGT1A1, UGT1A6a, and UGT1A9 showing substantial increases. These specific UGTs are widely acknowledged as the primary enzymes responsible for the glucuronidation process of Kae (Li et al. 2018). Consistent with our previous research, we also found that Kae has the capacity to upregulate the expression of the liver‐specific multidrug resistance‐associated protein 2 (MRP2) (Li et al. 2022). As we all know that UGTs and efflux transporter MRP2 co‐regulate the in vivo exposure of Kae metabolites (Zheng et al. 2016), therefore, we conclude that long‐term Kae exposure could increase its absorption and circulation in vivo via inducing the expression of UGTs and transport proteins.

When we analyzed the data of CYPs, founding an interesting phenomenon of the opposite changes in expression of CYP7A1 and CYP27A1 after 15‐day Kae treatment (Figures 2 and 3). Multiple methods, including qRT‐PCR, western blotting, and LC–MS/MS methods, were applied to confirm that Kae treatment could decrease the expression of CYP7A1 and increase the expression of CYP27A1, respectively. Although the results of western blot and qRT‐PCR did not maintain complete consistency under the intervention of different doses of Kae, the overall upward trend was consistent. The reason for the inconsistency might be due to the non‐specificity of antibody. Furthermore, a closed correlation existed between protein amount (quantified by LC–MS/MS) and mRNA expression (Figure 3b). This also suggests that detecting the amount of CYP7A1 and CYP27A1 using LC–MS/MS method may be more accurate compared to the western blot method. In general, Kae can downregulate the CYP7A1 amount and upregulate the CYP27A1 expression. CYP7A1 and CYP27A1 are the main enzymes mediating the synthesis of BA in hepatocytes, suggesting the possible changes in BA composition. Hence, we further studied the changes of BA and found that the amounts of CBA (especially T‐CA and T‐DCA) were significantly increased after Kae treatment (Figure 4 and Figure S3).

Furthermore, relevant studies have demonstrated that the FXR plays a pivotal role in regulating BA metabolism by influencing the expression of key metabolic enzymes, including CYP7A1 and CYP27A1 (Lu et al. 2000; De Aguiar Vallim, Tarling, and Edwards 2013). Elevated levels of FXR can lead to a reduction in CYP7A1 expression through the induction of liver Shp expression (Chiang and Ferrell 2022). Our results showed that Kae could significantly increase FXR expression in the liver (Figure 5a–c), although the mRNA did not show statistical significance due to large individual differences among groups. However, the overall upward trend is consistent with the protein expression. The increased expression was similar to our previous report (Li et al. 2022). Molecular docking studies further substantiate these observations, confirming the interaction between Kae and human FXR through specific amino acid residues such as TYR361, TYR369, HIS447, and SER332. Notably, the binding site of Kae with FXR overlaps with that of the endogenous agonist (CDCA), as depicted in Figure 5d–g. Based on these collective results, we postulate that Kae may downregulate CYP7A1 expression while upregulating CYP27A1 expression through the activation of liver FXR. In addition to the observed increase in the CBA, we also examined the impact of Kae on hepatic enzymes involved in the CBA synthesis and fecal BSH activity. Our data indicate that Kae does not modulate the expression of these synthetic enzymes (Figure 5h) but could downregulate the BSH activity (Figure 5i,j). Collectively, these insights suggest that the observed increase in CBA levels is attributable to the reduced BSH activity, providing a novel perspective on the intricate regulatory mechanisms involving Kae, FXR, and BA homeostasis.

Given that BSH are derived from specific GM strains, we conducted an in‐depth analysis to compare the GM profiles after Kae treatment. Our findings indicated that Kae significantly altered the GM composition and enhanced its diversity. Particularly at the K100 dosage, Kae administration induced a notable shift in the Firmicutes‐to‐Bacteroidetes ratio in C57BL/6 mice, characterized by a substantial rise in Bacteroidetes and a concurrent decline in the Clostridium and Lactobacillaceae family. This shift is typically associated with obese individuals who manage their body weight through a fat‐restricted diet (Remely et al. 2015). Clostridium, Lactobacillaceae, and Enterobacteriaceae are important bacterium that can encode BSH and possess high hydrolytic capabilities (Song et al. 2019). Tian et al. also reported that berberine can reduce BSH activity by decreasing the abundance of Clostridium, thereby increasing the accumulation of T‐CA (Tian et al. 2019). These results indicate that Kae may reduce the activity of BSH enzyme by decreasing the abundance of these bacteria.

For GM further analysis, we found several beneficial bacteria (including *B. acidifaciens* , *B. choerinum* , *E. coprostanoligenes* , and *L. saccharolyticum*) were enhanced after being treated with Kae; however, certain pathogenic bacteria (such as *Alistipes*, *Citrobacter*, and *Helicobacter*) were effectively suppressed by the Kae. It is currently believed that the disease‐fighting mechanism of these probiotics primarily involves modulating the formation of short‐chain fatty acids and BAs to regulate intracellular signaling pathways (Then et al. 2020; Guo et al. 2022; Zheng et al. 2023). For example, *L. saccharolyticum* modulates the production of short‐chain fatty acids, particularly butyrate, to induce the accumulation of Treg cells and trigger the secretion of Il10 and Il12, thereby exerting anti‐inflammatory effects in the gastrointestinal tract (Grenda et al. 2022). On the contrary, the proliferation of pathogens has the potential to trigger, either directly or indirectly, a microenvironment conducive to inflammation, cell proliferation, and the progression of neoplastic conditions (Keefe 2016). For example, Helicobacter is a known pathogen, among which *Helicobacter pylori* can cause chronic inflammation and induce duodenal and gastric ulcers (Wroblewskiv 2010); in hypertensive patients, an increase in *Alistipes indistinctus* and *Alistipes putredinis* is positively correlated with systolic blood pressure, potentially by triggering intestinal inflammation and gut barrier dysfunction (Parker et al. 2020); *Alistipes putredinis* has a pathogenic role in colorectal cancer, possibly playing a leading role in disease regulation, or it may have an auxiliary or co‐inducing effect (Fu et al. 2024). Collectively, the findings indicate that Kae modulates the GM composition and function as a prebiotic to foster probiotics expansion and curb pathogen proliferation.

However, more in‐depth mechanistic studies are needed for further investigation. For instance, how Kae influences the upregulation of these probiotics, determining whether its effects are direct or indirect. Furthermore, it is essential to clarify which BAs are regulated by these altered bacteria. On the other hand, for Kae‐specific metabolic characteristics in vivo, whether its metabolic characteristics can regulate its pharmacological effects, and if so, whether reasonable combination therapies can uncover more efficient and less toxic treatment plans. These are all issues that need to be addressed in later research. Through a series of in‐depth studies, it is hoped that theoretical foundations can be provided for the more efficient clinical application of food‐derived Kae.

## 5 Conclusions

Kaempferol enhances its own metabolic circulation and BA synthesis by activating FXR to regulate the expressions of UGTs and CYPs enzymes. Furthermore, Kae significantly changes BA‐GM axis by inhibiting BSH activity, promoting the growth of probiotics, and inhibiting the growth of harmful GM. Collectively, our research indicates the promising prebiotic potential of Kae, highlighting its capacity to modulate the BA‐GM axis, thereby conferring nutritional and therapeutic advantages.

## Author Contributions

**Xiaoyan Li:** formal analysis (equal), funding acquisition (equal), methodology (equal), project administration (equal), writing – original draft (lead). **Guoxin Huang:** methodology (equal), supervision (equal), validation (equal), writing – review and editing (equal). **Imran Khan:** data curation (lead), formal analysis (equal). **Zhishan Ding:** validation (equal). **Wen Luan Wendy Hsiao:** writing – review and editing (equal). **Zhongqiu Liu:** funding acquisition (equal), supervision (equal).

## Ethics Statement

This study was approved by the Institutional Animal Care and Use Committee of the International Institute for Translational Chinese Medicine (IITCM_20180140).

## Conflicts of Interest

The authors declare no conflicts of interest.

## Supporting information


Appendix S1

## Data Availability

Supporting RNAseq data is deposited in the NCBI SRA (SRA:BioProject: PRJNA700452). Supporting Information can be found in the additional file.
