# Supplementary material for: The Prebiotic Effect of Kaempferol in Regulating Bile Acid Metabolism
Source: Food Sci Nutr. 2025 Feb 24;13(3):e70023. doi: 10.1002/fsn3.70023 (PMC11848346; doi:10.1002/fsn3.70023)
Supplement: Supplementary file 1 — Appendix S1 [file FSN3-13-e70023-s001.docx]

**Supporting Information**

**The prebiotic effect of Kaempferol in regulating bile acid metabolism**

Xiaoyan Li^1,2†*^, Guoxin Huang^4^^†^, Imran Khan^5^, Zhishan Ding^1^, W.L.Wendy Hsiao^3^, Zhongqiu Liu^2,3^

1 School of Medical Technology and Information Engineering, Zhejiang Chinese Medical University, Hang-zhou, Zhejiang, 310000, China.

2 International Institute for Translational Chinese Medicine, Guangzhou University of Chinese Medicine, Guangzhou, Guangdong, 510006, China.

3 State Key Laboratory of Quality Research in Chinese Medicine, Macau University of Science and Technology, Macau, 999078, China.

4 Clinical Research Center, Shantou Central Hospital, Shantou, China.

5 Department of Biotechnology, Faculty of Chemical and Life Sciences, Abdul Wali Khan University Mardan, KPK, Pakistan.

* Correspondence:

Doctor Xiaoyan Li, School of Medical Technology and Information Engineering, Zhejiang Chinese Medical University, Hangzhou, Zhejiang, 310000, China. E-mail: [lixiaoyan7755@163.com](mailto:lixiaoyan7755@163.com).

†: These two authors contributed equally to this work


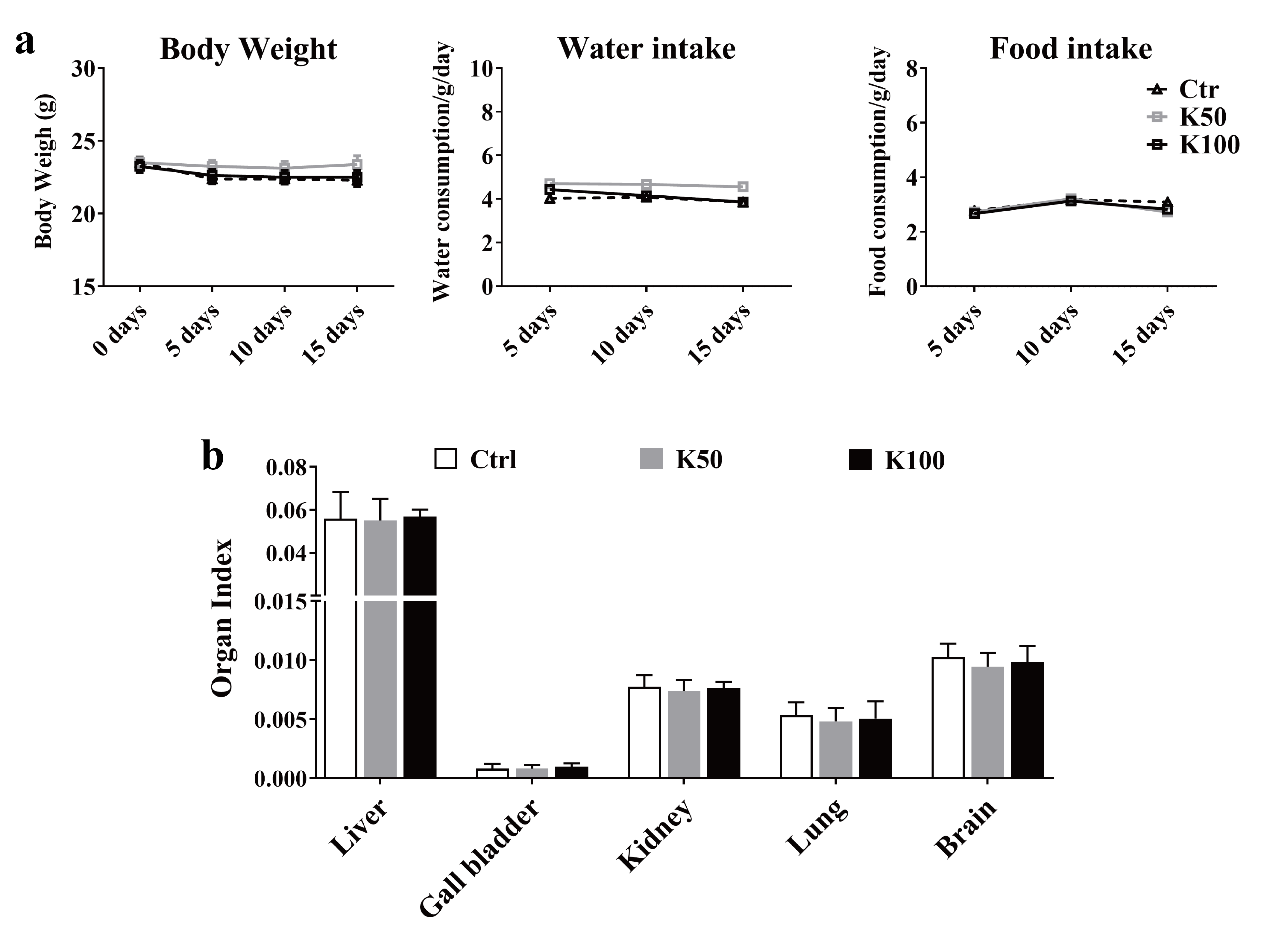


**Figure S1.** Effect of Kae on body weight, water intake, food intake and organ index in C57BL/6 mice. (a) Body weight, water intake and food intake. (b) Organ index. The data are presented as the mean ± SD values (n=8). *P* < 0.05 (*), *P* < 0.01 (**) and *P* < 0.001 (***) denote statistical significance for all statistical analyses. Ctr: C57BL/6 mice; K50: C57BL/6 mice treated with 50 mg/kg Kae; K100: C57BL/6 mice treated with 100 mg/kg Kae.


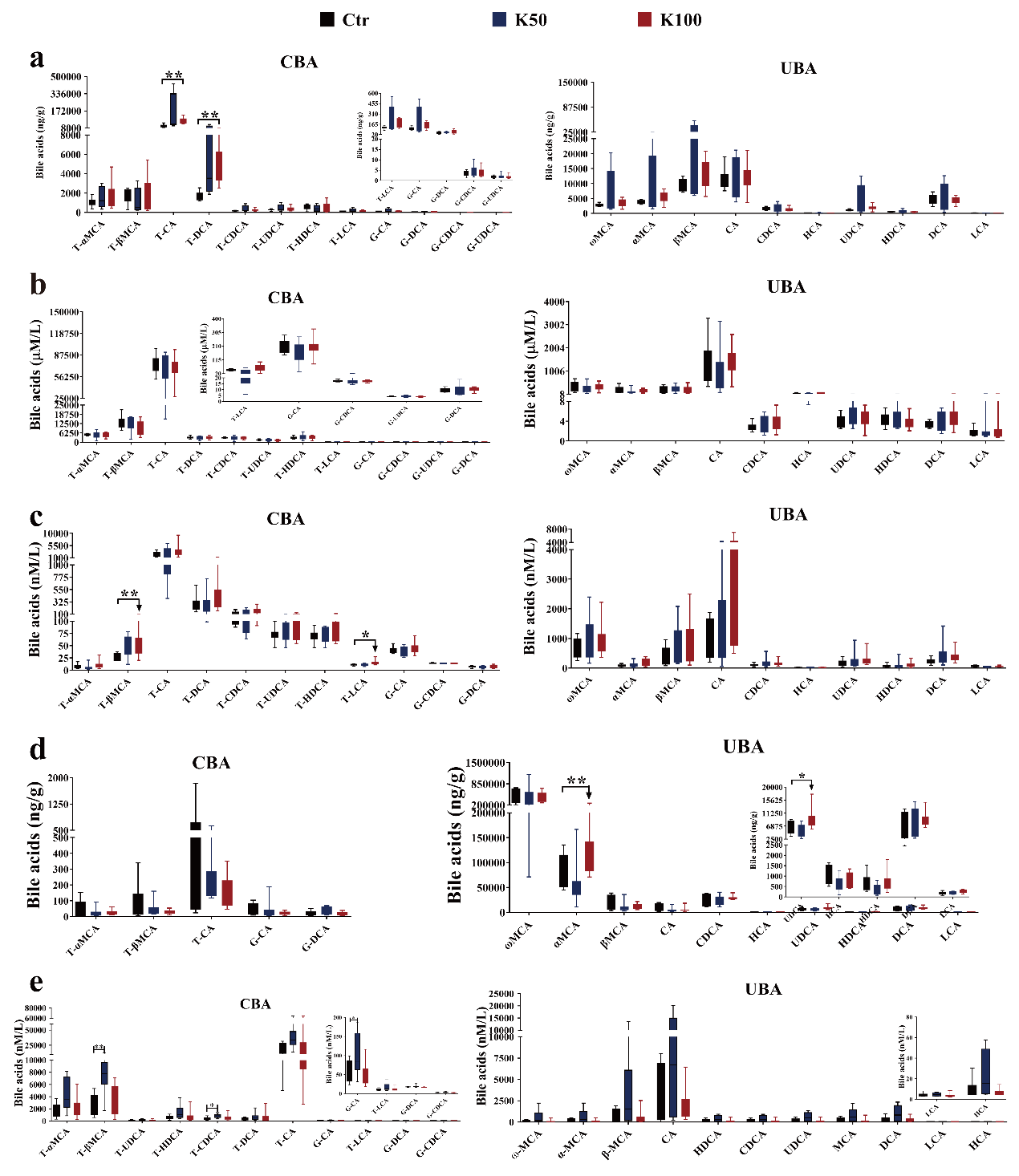


**Figure S2.** **Effects of Kae on BAs in C57BL/6 mice.** The amount of BA in liver tissue (a); in bile (b); in plasma (c); in feces (d) and in duodenal contents (e). The data are presented as the mean ± SD values (n=8). *P* < 0.05 (*), *P* < 0.01 (**) and *P* < 0.001 (***) denote statistical significance for all statistical analyses. Ctr: C57BL/6 mice; K50: C57BL/6 mice treated with 50 mg/kg Kae; K100: C57BL/6 mice treated with 100 mg/kg Kae.


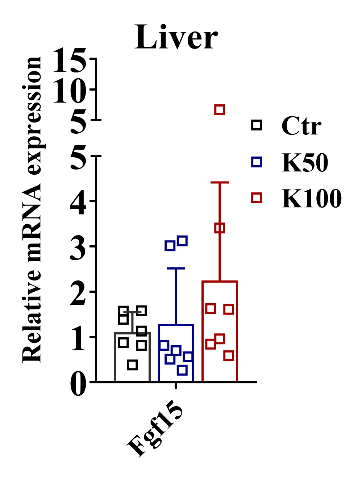


**Figure S3.** The mRNA expression levels of *Fgf15* in liver tissue after Kae treatment. The data are presented as the mean ± SD value, n=7. There are no statistical significances for *Fgf15* expression after Kae treatment.


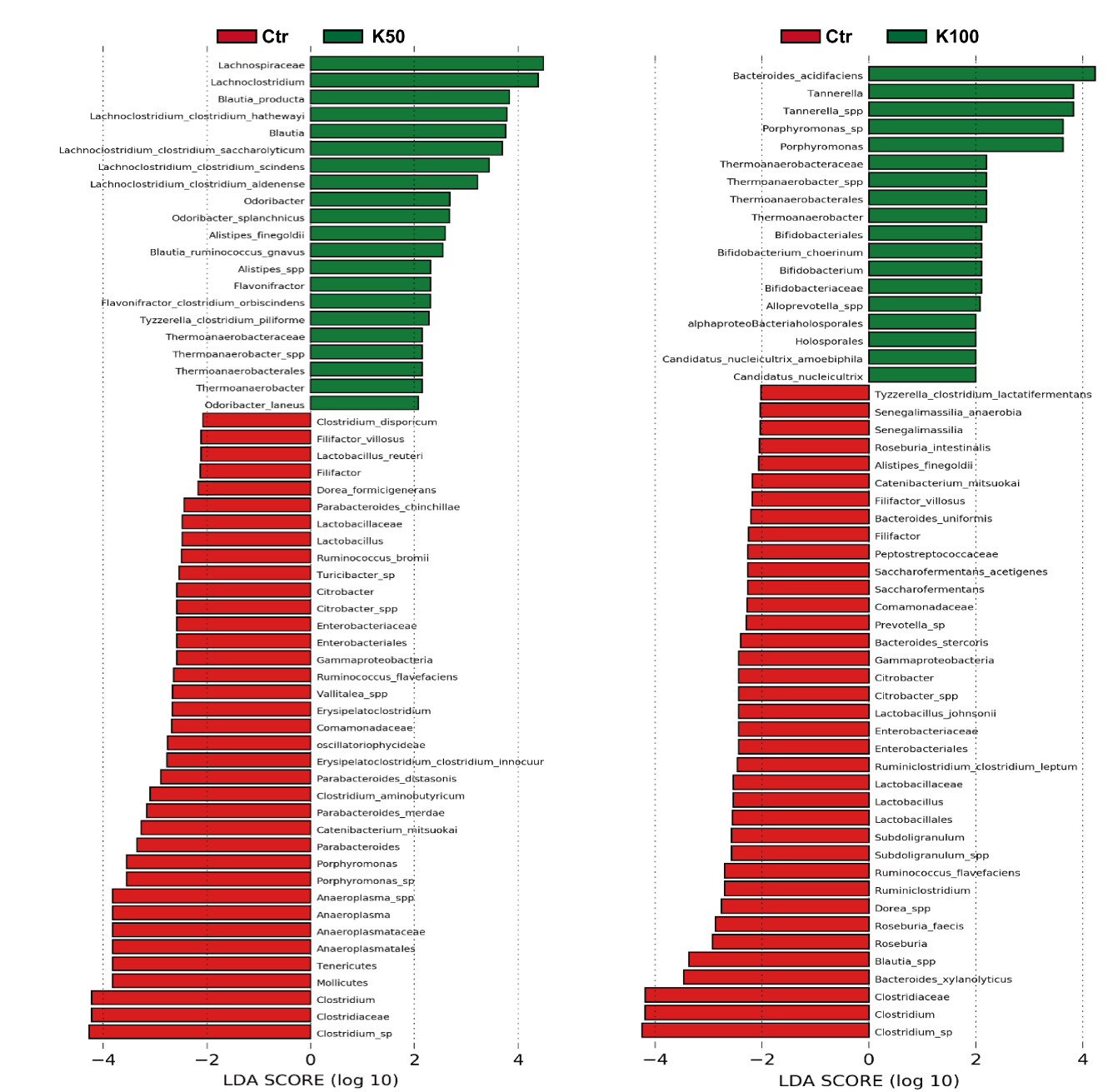


**Figure S4.** The prebiotic effect of Kae. The data are analyzed via linear discriminant analysis (LDA) method, n=8.


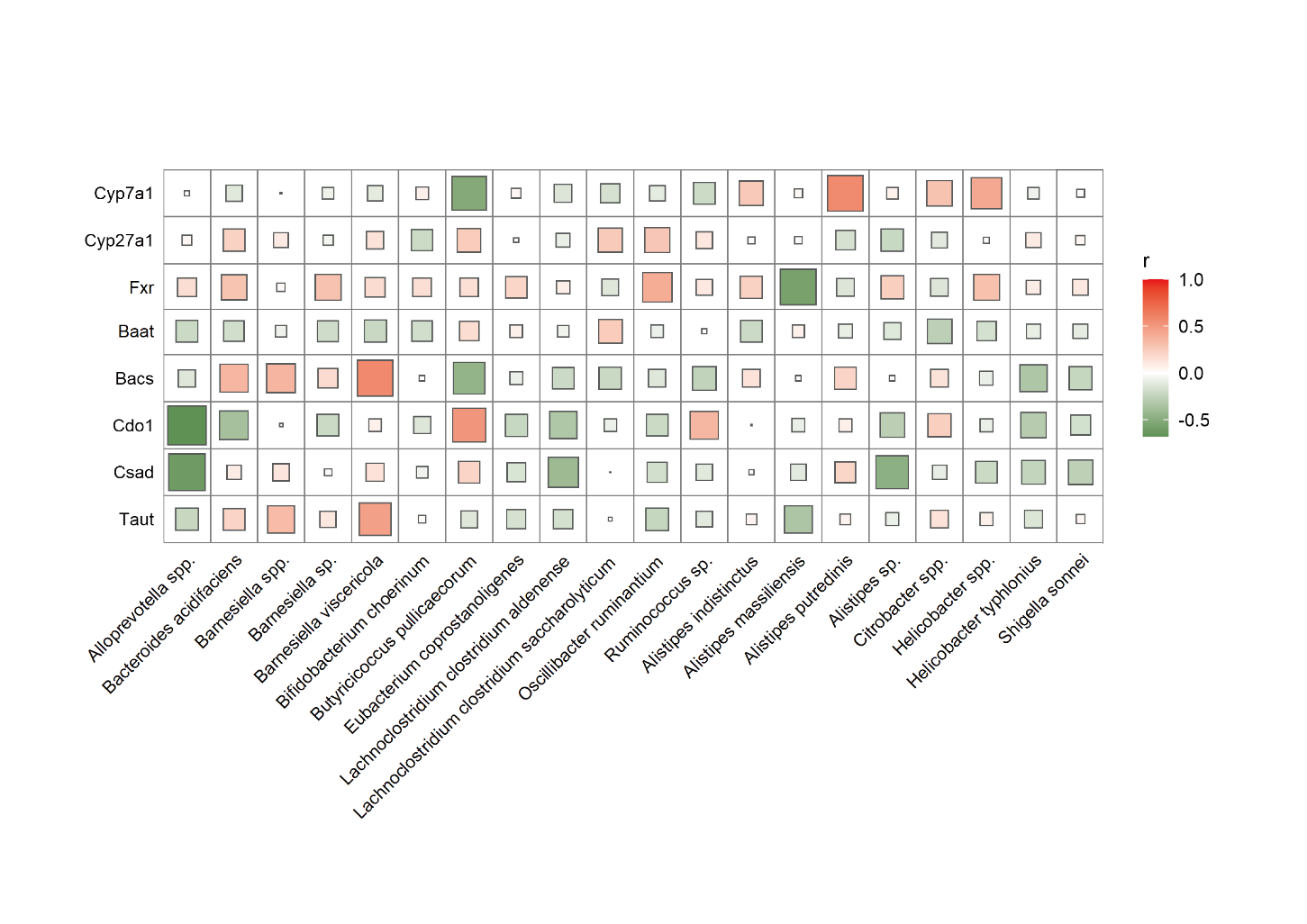


**Figure S5.** The correlation between the changed GM (including the probiotics and harmful bacteria shown in Figure 8) and the liver protein related to the BA synthesis and metabolism. The closer to red indicates a stronger positive correlation, while the closer to green indicates a stronger negative correlation. The larger the square in the grid, the more significant the correlation.

**Table S1.** PCR primer for target genes and *GAPDH* of mouse.

| **Target gene** | **Nucleotide sequence of primer (5’ to 3’)** | |
| --- | --- | --- |
|  | **Forward** | **Reverse** |
| ***Cyp7a1*** | GGGAATGCCATTTACTTGGATC | TATAGGAACCATCCTCAAGGTG |
| ***Cyp27a1*** | TACTCAGGAGACCATCGGCA | ATCCGGGAGTTTGTGGGAAC |
| ***Fxr*** | GTTCGGCGGAGATTTTCAATAAG | AGTCATTTTGAGTTCTCCAACAC |
| ***Shp*** | CCAGAATCCCTCCTGACTTTG | TGGAGATTGGTTGGTCCTTTC |
| ***Fgr15*** | AGACGATTGCCATCAAGGACG | GTACTGGTTGTAGCCTAAACAG |
| ***Baat*** | GGAAACCTGTTAGTTCTCAGGC | GTGGACCCCCATATAGTCTCC |
| ***Bacs*** | TCTATGGCCTAAAGTTCAGGCG | CTTGCCGCTCTAAAGCATCC |
| **Cdo1** | GACCAGAGAACAGGGCATAAA | CCAGTGACCCTGAAGTTGTAAA |
| ***Csad*** | AATAAGCAGTCCAGCCCTATTC | GAGTCAGCCATCAGGATGAAA |
| ***Tαut*** | GGCCAGTGGACCAAATGATA | TGTCAAGAGGCAAGGAAGAATAG |
| ***Gapdh*** | TGTGTCCGTCGTGGATCTGA | TTGCTGTTGAAGTCGCAGGAG |

**Table S2.** Pharmacokinetic parameters of Kae and its metabolites in C57BL/6 mice before or after 14-day Kae treatment with doses of 50 and 100 mg/kg. Data are presented as means ± SD (n = 4). The data with green font shows differences before versus after Kaempferol-treated group. The symbol of ** means a significant difference, *P* < 0.01.

|  | **Group** | **T_max_ (min)** | **C_max_ (μM)** | **AUC_0→t_ (min•μM)** | **AUC_0→∞_ (min•μM)** | **T_1/2_ (min)** | **MRT_0→∞_ (min)** |
| --- | --- | --- | --- | --- | --- | --- | --- |
| **Kae** | K50-D0 | 13.00±9.89 | 2.48±1.99 | 292.41±32.64 | 447.65±1.10 | 425.09±65.21 | 610.24±126.42 |
|  | K50-D14 | 5.00±1.73 | 10.44±13.39 | 414.31±266.97 | 525.28±193.40 | 456.20±446.02 | 562.64±537.36 |
|  | K100-D0 | 11.66±8.50 | 18.19±20.58 | 916.91±909.6 | 1046.95±960.05 | 279.47±137.24 | 380.62±228.11 |
|  | K100-D14 | 8.00±3.46 | 18.16±8.10 | 693.23±186.08 | 718.22±173.35 | 149.34±43.98 | 149.21±47.72 |
| **K-3G** | K50-D0 | 12.00±0.00 | 51.79±17.50 | 3062.77±315.67 | 3123.04±238.13 | 306.24±327.41 | 92.42±46.91 |
|  | K50-D14 | 12.00±0.00 | 107.67±45.91 | 4821.16±2778.42 | 4904.55±2865.45 | 197.5±106.98 | 81.89±14.40 |
|  | K100-D0 | 23.33±5.77 | 121.72±101.73 | 8623.57±6469.43 | 8652.26±6481.92 | 103.42±3.13 | 91.14±25.71 |
|  | K100-D14 | 18.50±8.54 | 226.93±48.92 | 11458.65±4273.08 | 11551.14±4257.38 | 185.16±151.32 | 74.60±11.85 |
| **K-7G** | K50-D0 | 9.00±4.24 | 6.69±3.66 | 400.57±97.26 | 402.96±96.95 | 89.81±5.67 | 89.51±18.18 |
|  | K50-D14 | 12.00±0.00 | 20.63±13.53 | 952.63±698.57 | 979.37±725.15 | 192.08±97.63 | 110.09±31.52 |
|  | K100-D0 | 9.66±9.07 | 71.16±70.93 | 2629.96±2149.81 | 2640.85±2152.95 | 115.32±13.03 | 82.61±34.91 |
|  | K100-D14 | 12.00±0.00 | 89.98±22.03 | 2997.17±1476.75 | 3003.92±1478.63 | 85.82±3.38****** | 61.62±6.72 |
| **K-7S** | K50-D0 | 90.00±42.42 | 2.31±0.46 | 603.48±36.26 | 694.35±74.01 | 270.63±112.71 | 317.34±39.33 |
|  | K50-D14 | 98.40±64.28 | 3.19±1.34 | 841.28±325.02 | 970.64±329.41 | 255.10±53.60 | 337.62±55.58 |
|  | K100-D0 | 86.66±57.73 | 17.10±22.82 | 1871.00±1771.33 | 2026.03±1918.60 | 230.44±102.44 | 318.28±147.08 |
|  | K100-D14 | 12.00±0.00 | 7.00±1.07 | 1606.61±286.20 | 1779.07±336.11 | 203.74±29.80 | 275.57±19.20 |
